# Optic pathway glioma and the sex association in neurofibromatosis type 1: a single-center study

**DOI:** 10.1186/s13023-021-02121-8

**Published:** 2021-11-22

**Authors:** Anne Munk Henning, Mette Møller Handrup, Sia Mariann Kjeldsen, Dorte Ancher Larsen, Cecilie Ejerskov

**Affiliations:** 1 Center for Rare Diseases, Department of Pediatrics and Adolescent Medicine, Aarhus University Hospital, Palle Juul-Jensens, Boulevard 99, 8200 Aarhus N, Denmark; 2 Department of Ophthalmology, Aarhus University Hospital, Palle Juul-Jensens Boulevard 167, 8200 Aarhus N, Denmark; 3 Department of Clinical Medicine, Aarhus University, Aarhus, Denmark

**Keywords:** Neurofibromatosis type 1, Optic pathway glioma, Sex characteristics, Ophthalmological screening

## Abstract

**Background:**

Low-grade optic pathway glioma (OPG) develops in 15–20% of children with neurofibromatosis type 1 (NF1). OPGs are symptomatic in 30–50% and one-third of these require treatment. A few studies have suggested female sex as a risk factor for visual impairment associated with NF1-OPG. This descriptive study investigated the correlation between NF1-OPG growth, sex and visual impairment.

**Method:**

We based our cross-sectional study on a systematic, retrospective data collection in a NF1 cohort of children and adolescents below 21 years of age followed at Center for Rare Diseases, Aarhus University Hospital, Denmark. For each patient with OPG a medical chart review was performed including demographics, ophthalmological examinations and magnetic resonance imaging (MRI) of OPG.

**Results:**

Of 176 patients with NF1 (85 females, 91 males), we identified 21 patients with OPG (11.9%) with a preponderance of females, *p* = *0.184*. Eight females (62%) and one male (13%) had visual impairment at the last ophthalmological evaluation. Five out of 21 children with OPG (24%) underwent diagnostic MRI because of clinical findings at the ophthalmological screening. Nine children (43%) had symptoms suggestive of OPG and seven (33%) experienced no OPG-related symptoms before the diagnostic MRI. Of eight children diagnosed with OPG ≤ two years of age, one had visual impairment. Of 13 children diagnosed > two years of age, eight had visual impairment*;* in each group, four of the children were treated with chemotherapy. The study suggested no correlation between NF1-OPG growth and sex.

**Conclusion:**

Our data suggest sex as a risk factor for visual impairment, while an OPG diagnose ≤ two years of age was a protective factor for visual impairment. Females with NF1-OPG had a higher prevalence of visual impairment outcome compared to males. Interestingly, our data also suggest a better response to treatment in children with OPG diagnosed ≤ two years of age compared to older children. The findings in our study suggest sex as a potential prognostic factor for visual impairment.

## Background

Neurofibromatosis type 1 (NF1) is a complex autosomal dominant disorder caused by germline pathogen variations in the *NF1* tumor suppressor gene [1, 2]. NF1 is a rather common rare disease, and the incidence is one case per 2500–3000 individuals worldwide [2, 3].

Approximately 15–20% of children with NF1 develop a benign, low-grade optic pathway glioma (OPG) [3, 4]. In the majority of children the OPG is diagnosed before seven years of age (mean 4.5 years) [5]. The OPGs are symptomatic in 30–50% of the children causing mainly vision loss and endocrine abnormalities. One-third of OPGs require therapeutic intervention due to visual impairment or increase of OPG on imaging [4, 5]. Treatment of NF1-OPGs is primarily reserved for patients with progressive clinical symptoms and commonly involves chemotherapy [6].

Optic pathway glioma can develop anywhere along the optic pathway [7–9]. OPGs are generally slow growing benign tumors, but individual tumors can have dramatic variability in their growth pattern with periods of rapid growth, prolonged quiescence or spontaneous regression [10–12]. The tumor growth may result in unilateral exophthalmus, visual impairment, visual field defect and ophthalmological findings such as an afferent pupillary defect, optic nerve atrophy, defects in color vision, strabismus, papilledema or precocious puberty [5, 13]. Magnetic resonance imaging (MRI) is the modality of choice for diagnostic purposes in NF1 patients with unexplained ophthalmological abnormalities [14–16].

Currently, our outpatient clinic at the Department of Ophthalmology, Aarhus University Hospital (AUH), performs an ophthalmological screening every six month until the age of eight years, and an annual screening from eight to 12 years of age. Ophthalmological examinations including age specific monitoring of NF1-OPG manifestations are important, as risk factors for visual impairment are a diagnosis of OPG below the age of two years, tumor involvement of the posterior optic tract and germline *NF1* pathogenic variations [3, 17, 18].

In July 2020, we performed a literature search in the databases PubMed and EMBASE to research if sex could be a potential risk factor for visual impairment. The inclusion criteria were: (1) Case reports or case series describing the potential role of sex as a clinically relevant modifier for symptoms caused by OPG in children with NF1, (2) studies researching the responsible etiology behind potential sex characteristics and OPG associated to NF1 and (3) reviews of NF1-OPG focusing on clinical manifestations and risk modifiers such as the sex of the patient. The search identified five articles: two clinical studies, two experimental studies and one article that included both a clinical and an experimental part [19–23]. Of the clinical studies, two studies presented a significant overrepresentation of females with NF1 developing OPGs [19, 20]. One study found that females were twice as likely to undergo brain MRI for visual symptoms and three times more likely to require treatment due to visual impairment than their male counterparts [21].

The aim of this study was to further investigate the correlation between NF1-OPG growth, visual impairment and sex to optimize screening guidelines for OPG in children with NF1.

## Methods

### The NF1-OPG cohort

We included children with NF1-associated OPG born between January 2000 and January 2020 referred to Center for Rare Diseases (CRD), Department of Pediatrics and Adolescent Medicine, AUH, one of two Danish national specialist centers for NF1 with West Denmark as catchment area. The center monitors patients with NF1 from infancy until older age, regardless of severity of disease and socioeconomic background as the public Danish healthcare system is tax-financed. The children met the clinical diagnosis of NF1 according to established international criteria [24].

### Protocol

We conducted a small cross-sectional study based on retrospective data from the medical charts of patients with OPG. Patients were included if they had a confirmed clinical diagnosis of NF1, a follow-up at CRD after two years of age and OPG. An initial screening of the medical charts was performed to identify the patients for inclusion. A medical chart review was then performed, and data were collected for each patient with a diagnosis of OPG. The data included information about demographic characteristics: sex, age at study inclusion, age at OPG diagnosis, indication for OPG diagnostic MRI, OPG location, ophthalmic examinations evaluating developmental change of visual impairment, MRI descriptions on tumor growth and potential treatment.

We defined the children’s visual acuity based on their inferior eye to evaluate the effect of the OPG on sight. Visual acuity was divided into two categories: Normal vision and Visual impairment. Visual impairment was defined as a Snellen visual acuity below 0.63 for children up to five years of age, while children six years or older had Visual impairment with a Snellen visual acuity below 0.80. A visual acuity above 0.63 and 0.80 for age, respectively, was defined as Normal vision. A difference in two lines or more on the eye chart between the inferior and the superior eye was categorized as Visual impairment.

The location of the OPG was defined from the diagnostic MRI, and progression tendency was defined from the last MRI description on OPG growth.

Informed consent was obtained from included patients and parents in cases of under aged patients with a diagnosis of OPG. Only data from medical charts on the included patients were available. Personal identifiable data have been omitted from this study. The study was approved by the Danish Data protection Agency. According to the Ethics Committee of Denmark, no approval was needed for this retrospective database investigation.

### Data analysis

A data project was created in the Research Electronic Data Capture (REDCap) to collect and manage data. REDCap is a secure web-based platform designed to virtually capture data for research studies and can provide automated export functions for data downloads internationally [25, 26].

### Statistical analyses

Descriptive statistics for the main variables of interest were calculated using proportions for categorical data and means, medians and ranges for continuous data. Statistical analyses were performed in Maplesoft. The Chi square test was used if all of the expected numbers were higher than five. If an expected number was less than five, statistics were not calculated because of the small data collection and the risk of chance findings. A *p* value < 0.05 was considered statistically significant.

## Results

### The CRD-OPG cohort

**Fig. 1:**
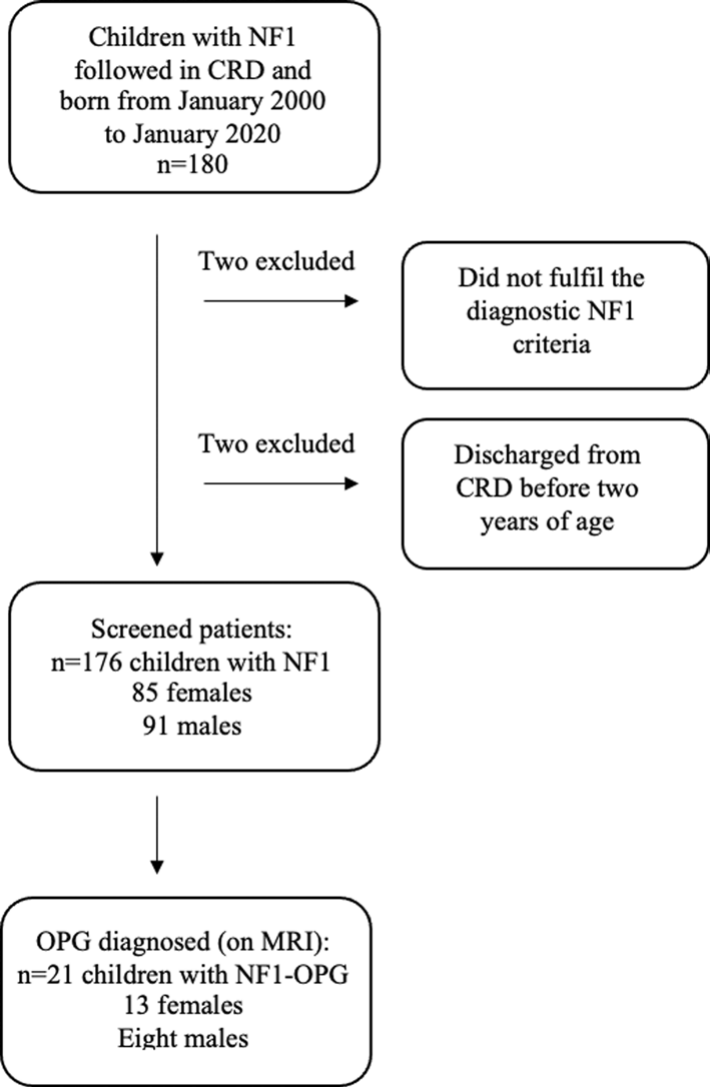
The initial screening of the patient cohort and final patient inclusion. CRD: Center for Rare Diseases, OPG: Optic pathway glioma, MRI: Magnetic resonance imaging

A total of 180 patients were screened and 176 patients with NF1 (48.3% females and 51.7% males) were eligible for inclusion (Fig. 1).

Table 1 presents the initial screening of the CRD patients and the included CRD-OPG cohort. In total 21 children (11.9%) were identified with OPG and included in the study; thirteen females (15.3%) and eight males (8.8%) out of the 176 children had OPG. There was a preponderance of females with OPG, *p* = *0.184*. The follow-up time was calculated from the date of the OPG diagnosis to the date of the last ophthalmological evaluation.

**Table 1:** Demographics of children with OPG

	Females	Males	Total
Children with NF1, n (%)	85 (48.3)	91 (51.7)	176 (100.0)
Median age at study inclusion, year, range	12 [6–20]	10 [5–18]	11 [5–20]
*Age at OPG diagnosis*			
≤ 2 years old, year (%)	4 (31)	4 (50)	8 (38)
2–5 years old, year (%)	4 (31)	1 (13)	5 (24)
≥ 5 years old, year (%)	5 (39)	3 (38)	8 (38)
Median age, year, range	5 [1–13]	3 [2–7]	4 [1–13]
Follow-up from OPG diagnosis			
Median duration, year, range	7 [2–13]	6 [0–16]	7 [0–16]

*OPG: Optic pathway glioma

### The diagnostic MRI—indications

Table 2 presents the different indications for diagnostic MRIs. Five out of 21 children with OPG (24%) underwent a diagnostic brain MRI based on clinical findings at the ophthalmological screening; in three of the four children with a unilateral change in color or shape of the optic disc, the diagnostic MRI showed an OPG in the opposite eye. In nine out of the 21 children (43%) the indication for a diagnostic MRI was other symptoms suggestive of OPG. The symptoms were most often noticed by the parents. Five of the children with other symptoms suggestive of OPG were diagnosed with NF1 and OPG at the same time, and they did not follow the ophthalmological screening program before their MRI. Seven children (33%) experienced no OPG-related symptoms before MRI was performed.

**Table 2:** Indication for OPG diagnostic MRI

	Females n: 13 (%)	Males n: 8 (%)	Total n: 21 (%)
*Ophthalmological screening*			
Total, n (%)	3 (23%)	2 (25%)	5 (24%)
Optic disc*, unilateral	2	2	4
Reduced visual acuity	1		1
*Other symptoms suggestive of OPG*			
Total, n (%)	5 (38%)	4 (50%)	9 (43%)
Change in visual behavior	1	1	2
Strabismus	1		1
Exophthalmus	1	1	2
Macrocephaly		2	2
Hydrocephalus	1		1
Growth retardation	1		1
*No OPG-related symptoms*			
Total, n (%)	5 (38%)	2 (25%)	7 (33%)
Routine brain MRI at diagnosis or as part of surveillance	5	2	7

OPG: Optic pathway glioma

MRI: Magnetic resonance imaging

*Change in color (pale) or shape of the optic disc

### OPG location, visual outcome, progression and treatment

The OPG location, visual outcome, progression and treatment are presented in Table 3. In females, the OPG mostly involved the optic nerves (62%); in males, the chiasma or anterior/posterior pathway were mostly involved (63%). Of the 11 patients with OPG of the optic nerve, four (36%) had visual impairment. Five (50%) had visual impairment of the 10 patients with OPG involving chiasma ± anterior/posterior pathway.

**Table 3:** OPG Location, treatment, progression and visual outcome

	Females n: 13 (%)	Males n: 8 (%)	Total n: 21 (%)
*Location*			
Optic nerves	8 (62%)	3 (38%)	11 (52%)
Chiasma ± anterior/posterior pathway	5 (38%)	5 (63%)	10 (48%)
*Visual outcome*			
Normal	5 (38%)	7 (88%)	12 (57%)
Visual impairment	8 (62%)	1 (13%)	9 (43%)
*Progression (tendency)*			
Stable	9 (69%)	3 (60%)	12 (67%)
Radiological regression	2 (15%)	2 (40%)	4 (22%)
Radiological progression	2 (15%)		2 (11%)
Treatment*			
Untreated	7 (54%)	6 (75%)	13 (62%)
Treated	6 (46%)	2 (25%)	8 (38%)

OPG: Optic pathway glioma

*The mainstay of treatment: Chemotherapy

Eight of the female children (62%) were categorized as having visual impairment at their latest ophthalmological evaluation; this was only found in one (13%) of the male patients. In two females, tumor progression was described at the latest MRI. Radiological tumor progression was not found in any of the male children, but data on three males (38%) were missing since only on MRI had been performed.

Generally, treatment was initiated if ophthalmological findings showed decreased visual acuity and/or radiological evidence of growth, although the approach varied. In total, six females (46%) and two males (25%) were treated with a standardized chemotherapy regimen according to the then international protocol on OPG. Of the eight treated patients, one girl and one boy required radiation and one girl required surgery in addition to chemotherapy and targeted therapy (MEK-inhibitor Trametinib). Radiation, surgery and targeted therapy were initiated due to tumor progression identified radiologically or decreased visual acuity after chemotherapy was completed.

Treatment was required in four children diagnosed > two years of age. They started treatment at the age range from four to 13 years, and all had visual impairment at the last ophthalmological evaluation. Eight children were diagnosed with OPG ≤ two years old; five were diagnosed without having any symptoms suggesting OPG, one was diagnosed because of change in the optic disc but had OPG on the opposite eye, and two children experienced symptoms related to their tumors (hydrocephalus and exophthalmus). Among the eight children ≤ two years old at diagnosis, one was defined as having visual impairment, whereas eight out of 13 patients > two years of age at their OPG diagnosis were found to have visual impairment. Children diagnosed ≤ two years of age had 8.7 years’ follow-up, and our data suggest that they were less likely to have visual impairment than children diagnosed > two years of age. Four of the children diagnosed with OPG ≤ two years of age required treatment; three started treatment at the age range two to four years and had normal visual acuity at the last ophthalmological evaluation and one started treatment at the age of seven years and had visual impairment at the last ophthalmological evaluation.

## Discussion

This study is the first to investigate a potential association between NF1-OPG growth, visual impairment and sex within a Danish NF1 cohort. Firstly, our data suggest a correlation between the sex and visual impairment in patients below 21 years of age with NF1-OPG as females were more likely to have visual impairment than their male counterparts in our cohort. This is in line with a study by Diggs-Andrews et al [21]. Within our NF1-cohort, our results suggest that females with OPGs could require treatment more often than the males. However, our cohort was small, and chance findings are possible. OPG occurred more often in females compared to the males in our cohort; this difference was not significant. Furthermore, our study did not find that the OPG location played an important role in dictating the visual acuity. Tumor progression was only found in two females; however, radiological data were not available for three males, which made it difficult to identify potential associations to NF1-OPG growth.

Secondly, our data suggest that children ≤ two years of age were less likely to have visual impairment despite a longer follow-up time. This is in contrast to previous literature reporting a diagnosis of OPG before the age of two years as a risk factor for vision loss [3, 18, 27]. However, it has been suggested that the focus of most previous reports has been on radiographic changes and treatment strategies [28]. Still, in our study the children diagnosed ≤ two years of age were not identified at visual acuity examinations, which seem to be the most reliable screening test and risk factor for visual impairment [9, 29–31]. This is likely to bias the results and could explain why they had a better visual outcome compared to the older children in the cohort. Unfortunately, it is difficult to examine the visual acuity in young children and MRI is more easily performed on clinical suspicion, which could lead to detection of more asymptomatic OPGs. Still, screening baseline brain MRI evaluations in children with NF1 are not advocated, as the detection of OPG rarely changes management in the absence of symptoms.

Thirdly, within our cohort five of the OPGs were diagnosed due to clinical findings at the ophthalmological screening; four cases were due to change in the color or shape of the optic disc. Interestingly, three of the four children had OPG on the opposite eye than expected from the screening, which could question the accuracy and sensitivity of the ophthalmological screening methods and strategy. Moreover, whether these ophthalmological findings were due to symptomatic OPGs is questionable and if the aim of the screening was to detect all OPGs MRI would be the method of choice [15, 32]. Visual acuity testing can be difficult and unreliable in young children, and the child’s ability to cooperate is crucial. Still, literature supports that visual acuity testing is the most reliable indicator of a symptomatic OPG [5, 30, 31]. A recent study by Kinori et al. found not only visual acuity, but also optic disc appearance (pale or edematous) as strong predictors of poor visual outcomes in 45 children with NF1-OPG [28]. This was not shown in our study and may be due to the lack of accuracy of the ophthalmological screening methods or coincidental due to the small cohorts in both studies. Hence, more research is needed on reliable indicators of a symptomatic OPG.

Our literature search identified three articles on exploring the pathogenesis behind the possible sexual dimorphism [21–23]. Fisher, M. J., et al. studied visual outcomes in children with NF1-OPG following chemotherapy [33]. They did not find that sex was a prognostic factor for visual outcome in the treated population, but they reported a disproportionate number of females with OPG requiring treatment compared to the males, *p* = *0.0075* [33]. This might indicate that there is no correlation between sex and response to treatment and that females might have worse OPG symptoms compared to males. This sexual dimorphism has been explored in *Nf1* mutant mice, where only female mice presented reduced visual acuity from their OPG despite equal tumor volumes and proliferative indices [21]. Research has suggested that the dimorphism can result from estrogen activation of microglia in female *Nf1* mice [23]. Another study found a sex specific role for cAMP regulation in gliomagenesis in patients with NF1, specifically identifying ADCY8 elevating the OPG risk in females and decreasing risk in males^22^. The sexual dimorphism is in line with what was found in our study.

Interestingly, our study showed that in the treated children diagnosed with OPG ≤ two years of age only one child had a visual impairment at the last ophthalmological examination (25%). The treated children diagnosed with OPG > two years of age all had visual impairment (100%). The three treated children with normal vision all started treatment at four years of age or earlier, whereas the treated children with visual impairment started treatment between four and 13 years of age. This could indicate that treatment response is associated with the age of the patient and highlights the importance of age-specific monitoring of NF1-OPG manifestations. Further research is needed to investigate the potential correlation.

Our retrospective study has some limitations. As the study involves patients with a rare disease, the patient population was small which limited the statistical analyses. In small studies, there is a risk of the results to be chance findings. The OPG frequency of 11.9% is a cross-sectional proportion as some children can still develop OPG in the future or may already have an asymptomatic OPG. It would have been interesting to view the numbers and results of cerebral MRI in the non-OPG patients within our cohort, but due to ethics of the study we were only able to review and use data from the medical charts of patients with a diagnosis of OPG. The number of patient non-appearances at appointments caused missing data in our cohort resulting in a risk of limitation to the study result. Furthermore, five patients were diagnosed with OPG at the same time as diagnosed with NF1; therefore, they did not follow the ophthalmological screening program. If they had been enrolled in the ophthalmological screening program, the OPG might have been detected at an earlier stage, and their visual outcomes could have been different. It was also difficult to register tumor progression due to the lack of consensus regarding the specific interval of neuroimaging of already identified OPGs. The tumor sizes were not examined and therefore not available in this study, but the association between size and visual impairment would be interesting for future studies. The ophthalmological examinations used different tools to evaluate the visual acuity in accordance with the age of the child, which could affect the result of their last ophthalmological examination and our definition of Normal or Visual impairment. Furthermore, the screening strategy in OPG and indication of MRI varied among physicians.

## Conclusion

In conclusion, our study suggests sex as a risk factor for visual impairment, while an OPG diagnosis ≤ two years of age was a protective factor for visual impairment. We found that females with NF1-OPG had a higher prevalence of visual impairment outcome compared with males, this was in accordance with existing literature. Furthermore, our study found an OPG prevalence of 11.9% in patients below 21 years in our children and youth cohort with a preponderance of females (15.3%). We found no correlation between NF1-OPG growth and sex. The findings in our study suggest sex as a potential prognostic factor for visual impairment. Interestingly, our data also suggest a better response to treatment in children with OPG diagnosed ≤ two years of age compared to older children. However, our cohort was small, and more research is needed to determine not only sex but also other potential risk factors for NF1-OPG associated visual impairment to optimize screening guidelines for OPG in children with NF1.

## Data Availability

Could be available from the corresponding author on reasonable request.
